# Differences in vegetative growth of two invasive hawkweeds at temperatures simulating invaded habitats at two altitudes

**DOI:** 10.1038/s41598-020-58914-7

**Published:** 2020-02-07

**Authors:** Kris French, Eva Watts

**Affiliations:** Centre for Sustainable Ecosystem Solutions, School of Earth, Atmospheric and Life Sciences, University of Wollongong, Wollongong, NSW 2522 Australia

**Keywords:** Invasive species, Population dynamics

## Abstract

*Hieracium pilosella* and *H. aurantiacum* are invading alpine regions in New South Wales, Australia. In a glasshouse experiment we investigated germination and growth rates of these two species at temperatures simulating the altitudes where invasions are occurring from autumn to spring. We measured germination rates, growth rates and the development of stolons and ramets using seedlings and plantlets from established plants. Germination was low in *H. aurantiacum* and unaffected by altitude or seed age. *H. pilosella* showed site to site variability in germination but had greater germination. No species produced flower spikes. Both species grew rapidly and put at least twice as much biomass into roots compared to shoots. *H. aurantiacum* could begin to produce stolons after 27 days and seedlings grew a little larger than for *H. pilosella*. *Hieracium aurantiacum* put significantly more resources into ramets, allocating between 4–15% of biomass. *H. pilosella* produced 2.6 stolons month^−1^, in contrast to 9.8 stolons month^−1^ for *H. aurantiacum*. Furthermore, plantlets from established plants had vastly different growth rates. Plantlets of *H. aurantiacum* produced 2.1 leaves day^−1^ from late summer to winter where *H. pilosella* was 3 times slower for the same period but faster following winter. Both species were able to maintain strong growth over cooler months suggesting hawkweeds have the capacity for fast growth in the invaded range under high nutrients and lower competition. *H. aurantiacum* is likely to be a more effective invader than *H. pilosella* spreading through stolons and the development of weed mats.

## Introduction

Hawkweeds (*Hieracium* spp. Asteraceae) are native to subalpine regions in Central Europe but form significant invasions in grasslands in USA^1^, Argentina and Chile^2,3^ and New Zealand^4,5^. Three species have invaded Australia, but at present, in the alpine regions of NSW, two of the species are in early stages of establishment; orange hawkweed (*Hieracium aurantiacum* L.) and mouse-ear hawkweed (*Hieracium pilosella* L.)^6^.

Both species are undergoing an eradication program in New South Wales, Australia, however, three aspects are of concern for the establishment of these invasive daisies if eradication fails. Firstly, both species are important weeds in other grasslands and, while global changes in climate might reduce overall distribution, there is no reason why they will not become widely established in the NSW alps and invade lower altitudes^7,8^. Secondly *H. aurantiacum* and *H. pilosella* can be apomictic^9–11^ and can therefore spread quickly with the establishment of single plants. Thirdly, both species are matt-forming, producing many stolons with ramets which allow genets to quickly take over available space in the landscape. Clonal plants are thought to improve resource capture as nutrients are redistributed between ramets and mother plants^12,13^. Clonal growth has been shown to play an important role in the spread of invasive species^14,15^. For example, populations of S*olidago gigantea* in the invaded range used clonal growth more than in the native range^16,17^. So, the speed at which hawkweed genets grow is an important factor in determining the priorities for management to prevent spread. Understanding differences in growth associated with temperature will yield information on growth at different altitudes. Furthermore, while *H. pilosella* invasion has been widely studied in invaded sites^e.g.^^18,19^, there is little information on *H. aurantiacum* (but see^20,21^).

The speed of germination, growth of individual plants and production of stolons and ramets all form important aspects in establishment, particularly for species that rely so heavily on clonal reproduction. For the climates in which these two species have invaded in the NSW alps, we sought to compare the rate of growth, of both seedlings and plantlets to understand potential establishment rates. Furthermore, we sought some understanding of germination rates from different populations grown at temperatures reflective of establishment sites.

## Methods

Germination and growth trials were undertaken from January - October 2018. Experiments were undertaken in incubators and growth cabinets which could be maintained at temperatures reflective of the altitudes that these species currently experience in Australia. Incubators were able to maintain low temperatures experienced in winter and had a PAR of 21 µmol m^−2^ s^−1^ which is similar to that experienced under snow. Growth cabinets had a PAR of 485 µmol m^−2^ s^−1^ which equated to that experienced on a cloudy day (510 µmol m^−2^ s^−1^).

Temperatures were set each month using average weather station data from around 1000 m (Hill Prison Camp 1006 m) or 1700 m asl (Perisher Valley AWS 1738 m) for each month. To calculate a typical day time temperature for any month, we identified the maximum temperature for each day within a month then took the midpoint between the highest and lowest temperature. We followed the same method to identify the night time temperature using minimum temperatures for each night of a month. Day and night length throughout all experiments reflected, and were adjusted to, the months we simulated.

### Germination

Germination of *H. aurantiacum* used seeds from three sites collected over the 2017/2018 summer (termed New) and older seeds from four sites (termed Old) that had been stored in paper bags at room temperature for up to four years. For each site, eight replicates of each of three temperature treatments were set up; temperatures replicating 1000 m, 1700 m, and 200 m asl. Each replicate consisted of 25 seeds placed in a petri dish with filter paper moistened with distilled water. In all, there were 144 petri dishes containing 3600 seeds of *H. aurantiacum*. Petri dishes in temperatures mimicking alpine temperatures were placed in incubators, while the *H. aurantiacum* trial at 200 m was placed in the glasshouse.

Due to the eradication program and resultant scarcity of *H. pilosella* only one site with new seeds collected over the recent summer and three sites with old seed collected in the last 5 years were available. These sites are within metres as there is only one invasion area near Charlotte Pass. As seed numbers were low we could only measure germination at temperatures mimicking 1700 m. The new seed site had only enough seeds for 7 petri dishes while the other sites had 8 petri dishes each. Thus, the *H. pilosella* seed trial contained 31 petri dishes and 775 seeds.

Petri dishes were checked for germination every two weeks and the experiment was terminated on 10^th^ July after just over 15 weeks. Tetrazolium testing of viability of the remainder of seeds in the petri dishes was attempted but without success as the hawkweed seeds are extremely small and seeds did not show any stain even when attempted on new seed material.

We use the term seedlings to be small plants grown from seed in this experiment. To avoid confusion in this study, we use the term ‘ramet’ as any attached plantlet that has grown on a stolon from its parent seedling or plantlet (See Growth of Seedlings below), while ramets that have been separated from stolons and are independent are identified as ‘plantlets’ (see Growth of Plantlets below).

### Growth of seedlings

As seeds germinated they were planted under the same conditions under which they were germinated, in a seedling plug before being transferred to small pots containing a mixture of general potting mix and coarse river sand (2:1). At the beginning of the seedling growth trial, 40 *H. aurantiacum* seedlings at the three-leaf-stage were potted up from seedling plug trays into small pots (130 mm diameter). From the 1000 m germination trial, 20 were transferred into a growth cabinet set to 1000 m conditions and another 20 from the 1700 m germination trial were transferred into a growth cabinet set to temperatures at 1700 m (April temperatures). Twenty *H. pilosella* seedlings were also transferred to the cabinet set at temperatures reflecting those at 1700 m altitude. The seedlings were watered twice a week and fertilized once a month with Osmocote Plus Organics all purpose liquid fertilizer (N: 15.4%, P: 0.0%, K: 6.0%). Plants were moved back into the incubators in June in order to experience winter temperatures.

After 188–189 days, plants were harvested. Stolons and leaves were separated from root, and new ramets were counted and separated. All plant parts were dried at 70 °C for 10 days and then weighed to give dry biomass.

### Growth of plantlets

**Figure 1:**
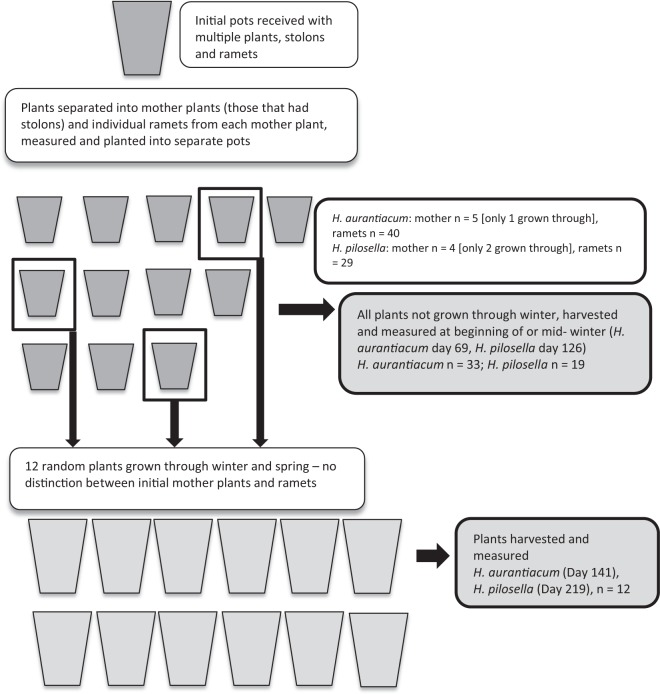
Experimental design for measuring growth of adult plants.

Two large pots of *H. aurantiacum* and one of *H. pilosella* were provided by the Department of Primary Industry. These pots contained multiple plants each with large numbers of stolons with numerous ramets attached. *H. pilosella* plants were at least 3 years old and grown from vegetative stock so three years represents a minimum age. *H. aurantiacum* pots were probably around two years old. Between 27^th^ February and 1^st^ March, ramets were separated and potted up into 130 mm diameter pots. In all, 45 *H. aurantiacum* and 33 *H. pilosella* ramets were separated into mother plant (those that had stolons emanating from them) and ramets (those who were found at the end of stolons emanating from a mother plant) (Fig. 1). Only ramets of at least 4 to 5 leaves were used; the rest discarded. Initially for *H. pilosella* there were 4 mother plants with an average of 4.75 ± 1.50 (s.d) stolons. There were 5 *H. aurantiacum* mothers with an average of 5.83 ± 9.02 (s.d) stolons. Only two of the mother plants from *H. pilosella* and one from *H. aurantiacum* grew and had leaves.

With these established plantlets, there was only room to grow each species under one altitude that best reflected its invasion conditions. Initially, *H. aurantiacum* plantlets were placed into a Thermoline growth cabinet set to temperatures reflective of 1000 m asl. *H. pilosella* plantlets were placed into a separate Thermoline growth cabinet set to temperatures reflective of 1700 m asl. At the beginning of winter due to limited space, we placed a random set of 12 pots of each species into incubators after repotting in 30 cm pots, to ensure winter temperatures were maintained and harvested all other plants (day 69 for *H. aurantiacum* and day 126 for *H. pilosella*, Fig. 1). Harvested plantlets were separated into above and below ground biomass, and individual ramets before drying and weighing.

On day 148 (26^th^ July 2018) *H. aurantiacum* plantlets were harvested as the 12 big plants had completely filled the pots. We continued to monitor for one week past our decision as they experienced some very high temperatures for 2 days at the end of our sampling when the cabinets failed to maintain set temperatures. Daytime temperatures reached 38–40 °C for a few hours on these days. Rather than harvest immediately, we harvested after a week (day 148) to observe any deaths following this event. Above and below ground dried biomass and individual ramet dry weight were recorded. The remaining 12 *H. pilosella* plantlets also received warmer than planned heating during July as well, but only increased to 24 °C which was not considered extreme for a warm day at this altitude. They were harvested on day 219 (9–11 October 2018) and above and below ground biomass as well as individual ramets were dried and dry weight recorded.

### Analysis

For *H. aurantiacum* the proportion of seeds germinated per petri dish was compared using a restricted maximum likelihood model with age and altitude as fixed factors and site nested within age. For *H. pilosella*, sites were compared as a fixed factor using a standard least squares model as there were no replicate sites nested within age and a lack of seeds meant we could only compare sites at the 1700 m altitude.

We compared the number of leaves, number of stolons and dry biomass measurements [above ground biomass, below ground biomass, total biomass, proportional mass in ramets and the root:shoot ratio] using mixed models with a binomial distribution and log link function. Stolon weight and leaves were included as above ground biomass while total weight included leaves, stolons, ramets and roots. Where significant effects were found, Tukeys HSD tests were used to identify where differences lay.

## Results

### Germination

**Figure 2:**
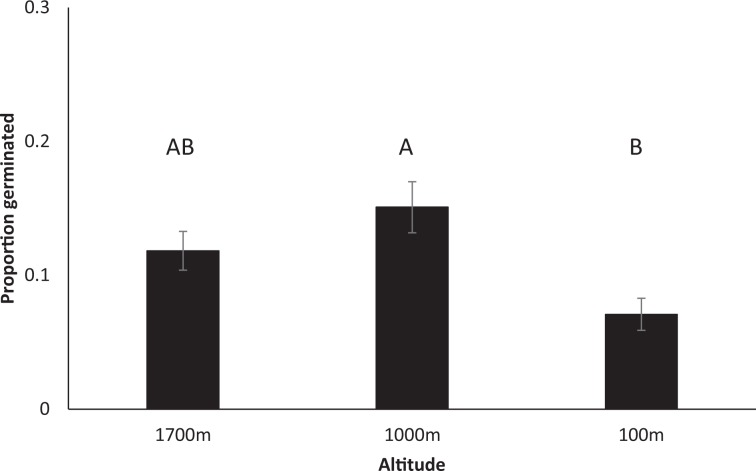
Proportion of *H. auranticum* seeds germinated in petri dishes at conditions representative of three different altitudes. Means based on n = 48.

**Figure 3:**
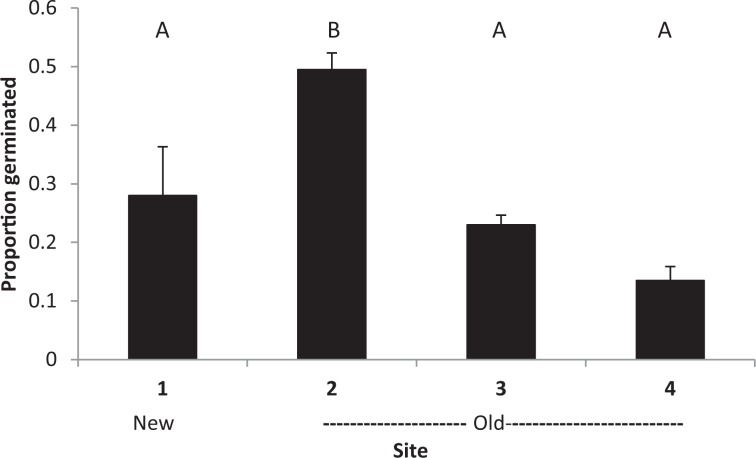
Proportion of *H. pilosella* seeds germinated in petri dishes from four sites at Mt. Koscuizsko. New seeds were fresh from the recent season’s reproduction while older seeds had been picked from earlier seasons. Means based on n = 48.

For *H. aurantiacum*, a greater proportion of seeds germinated at temperatures mimicking 1000 m than at 200 m with 1700 m being intermediate (F_2,9.30_ = 5.414, *P* = 0.028, Fig. 2). There was no difference in germination rate between old and new seeds (F_1,4.914_ = 0.571, *P* = 0.485) nor an interaction between the two main effects (F_1,9.30_ = 0.771, *P* = 0.490). Sites were highly variable with the proportion germinating ranging from 0.04 ± 0.01 to 0.31 ± 0.04 suggesting significant site to site variability in viable seed production. A greater proportion of seeds germinated for *H. pilosella*, with similarly high levels of variation in the proportion of seed germinating from different sites, but with no obvious relationship with age of seeds (F_3,27_ = 13.100, *P* < 0.0001, Fig. 3). Seeds of *H. aurantiacum* began germinating after 5 days reaching a maximum after a month, while seeds of *H. pilosella* began germinating after about 2 weeks and reached maximum after 5 weeks.

### Seedling growth

**Figure 4:**
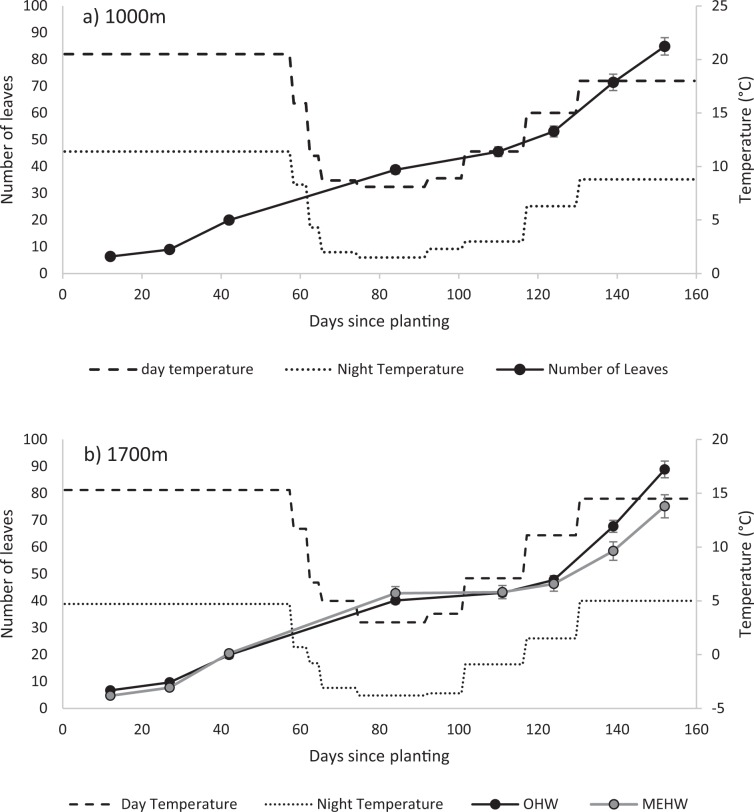
Average number of leaves (±s.e.) of seedlings potted from germinants grown at conditions representative of (**a**)1000 m and (**b**) 1700 m for *H. auranticum* (OHW) and *H. pilosella* (MEHW). There were no seedlings of *H. pilosella* at 1000 m. Lines reflect the day (dashed) and night (dotted) temperatures in the cabinets through the experiment.

**Figure 5:**
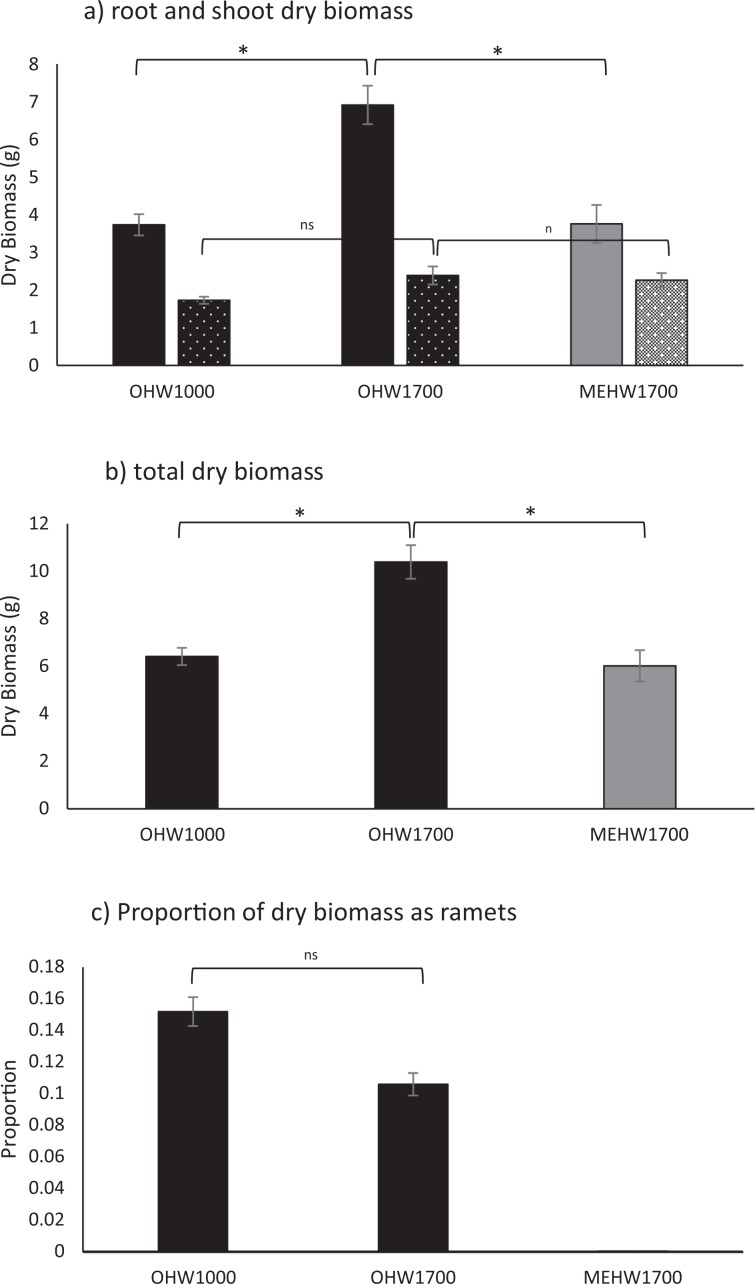
(**a**) Final average dry biomass per plant (±s.e.) of roots (solid) and shoots (pitted), (**b**) average total dry biomass including ramet weights, (**c**) proportion of total dry biomass that is new ramets, of seedlings at final harvest for *H. pilosella* (MEHW) and *H. aurantiacum* (OHW) at temperatures reflecting two altitudes (1000 m and 1700 m asl).

**Figure 6:**
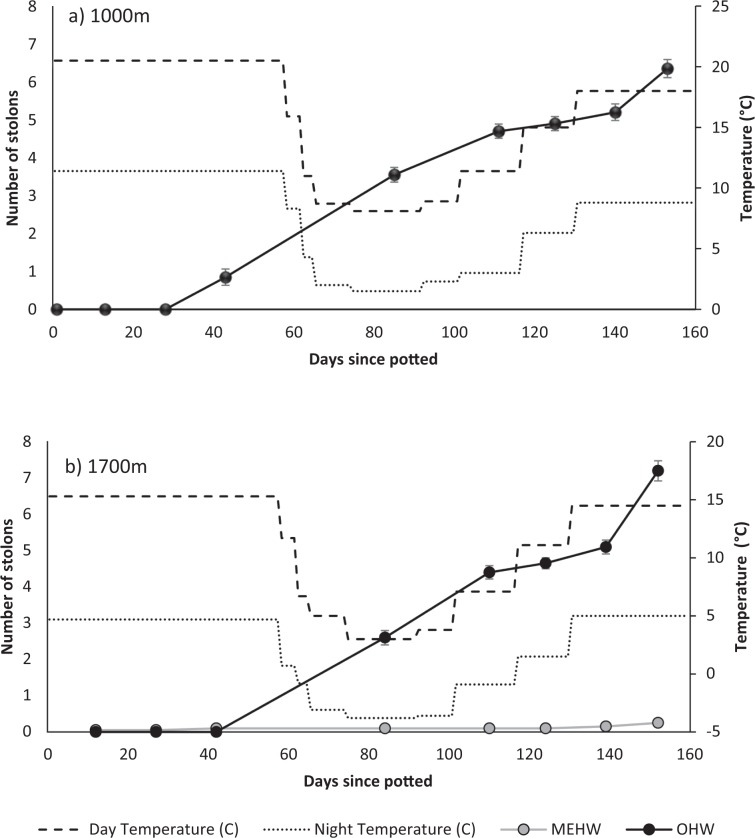
Average number of stolons (±s.e.) grown from seedlings potted from germinants grown at conditions representative of (**a**) 1000 m and (**b**) 1700 m of *H. aurantiacum* (OHW)and *H. pilosella* (MEHW). There were no seedlings of *H. pilosella* at 1000 m. Lines reflect the day (dashed) and night (dotted) temperatures in the cabinets through the experiment.

For *H. aurantiacum*, no seedlings survived in the glasshouses at 200 m asl. These experienced some very warm days and all seedlings died. Seedlings at other temperatures produced on average 86.9 ± 2.3 leaves over the experimental period with no difference in seedlings grown at temperatures reflecting different altitudes (1000 m vs 1700 m; χ_1_^2^ = 1.796, *P* = 0.180; Fig. 4). However, seedlings at temperatures reflecting 1700 m were larger than those at 1000 m (χ_1_^2^ = 19.020, *P* < 0.001; Fig. 5). The increase in dry weight was seen in the development of roots (χ_1_^2^ = 19.297, *P* < 0.001; Fig. 5) rather than above ground biomass (χ_1_^2^ = 2.115, *P* = 0.146; Fig. 5). *Hieracium aurantiacum* began producing stolons after 27 days from plantings at temperatures mimicking 1000 m but only after 40 days at when temperatures mimicked 1700 m although the proportion of dry weight as ramets (χ_1_^2^ = 2.906, *P* = 0.088; Fig. 5) and average number of stolons at the end of the experiment ($\bar{x}$ = 6.8 ± 0.2, χ^2^ = 1.067, *P* = 0.302; Fig. 6) did not differ with temperature. Root:shoot ratios did not differ between the two temperature regimes (OHW $\bar{x}$ = 2.4 ± 0.1, χ^2^ = 1.000, *P* = 0.317).

Seedlings of *H. aurantiacum* had more leaves ($\bar{x}$ = 88.9 ± 3.1) than *H. pilosella* seedlings ($\bar{x}$ = 75.2 ± 4.3) at 1700 m (χ_1_^2^ = 22.916, *P* < 0.001; Fig. 4b) and were significantly heavier (χ_1_^2^ = 23.576, *P* < 0.001; Fig. 5). This was due to an increase in roots (χ_1_^2^ = 18.976, *P* < 0.001; Fig. 5) but not above ground biomass (χ_1_^2^ = 0.067, *P* = 0.795; Fig. 5). Root:shoot ratios confirmed this change in resource allocation (MEHW $\bar{x}$ = 1.6 ± 0.1, χ^2^ = 5.254, *P* = 0.022). *H. pilosella* seedlings rarely produced stolons (and therefore ramets), averaging only 0.25 ± 0.06 stolons per plant (Fig. 6b).

### Plantlet growth

**Figure 7:**
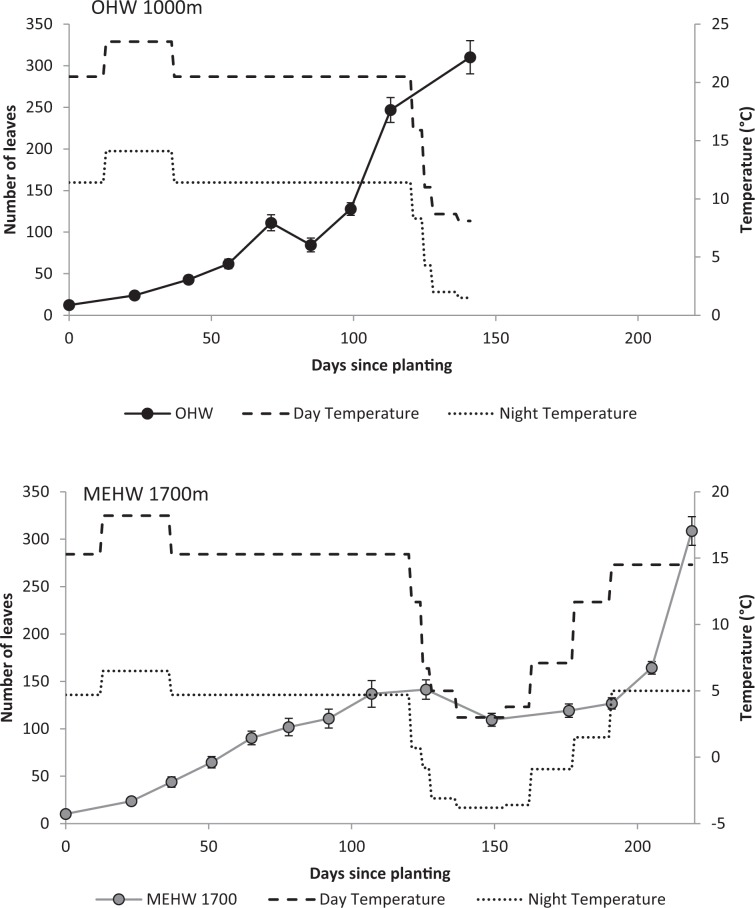
The number of leaves on established ramets through time of (**a**) *H. aurantiacum* (OHW) grown at conditions representative of 1000 m (**b**) *H. pilosella* (MEHW) grown at conditions representative of 1700 m. *H. aurantiacum* plants were terminated early as plants had filled pots. Lines reflect the day (dashed) and night (dotted) temperatures in the cabinets through the experiment.

**Figure 8:**
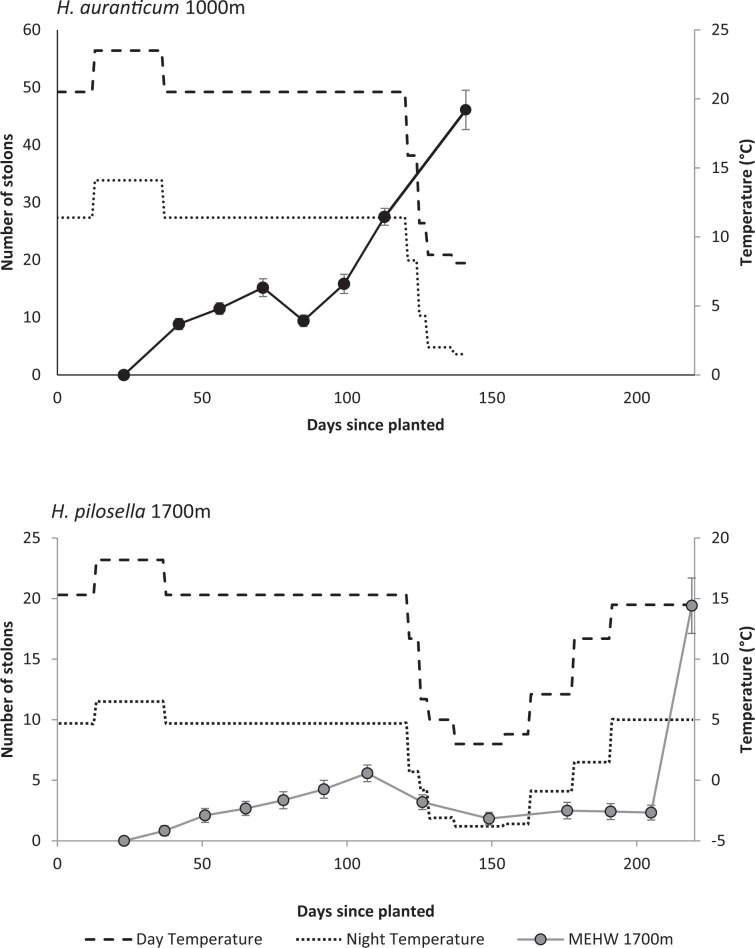
The number of stolons on established ramets through time of (**a**) *H. aurantiacum* (OHW) grown at conditions representative of 1000 m (**b**) *H. pilosella* (MEHW) grown at conditions representative of 1700 m. OHW plants were terminated early as plants had filled pots. Lines reflect the day (dashed) and night (dotted) temperatures in the cabinets through the experiment.

**Figure 9:**
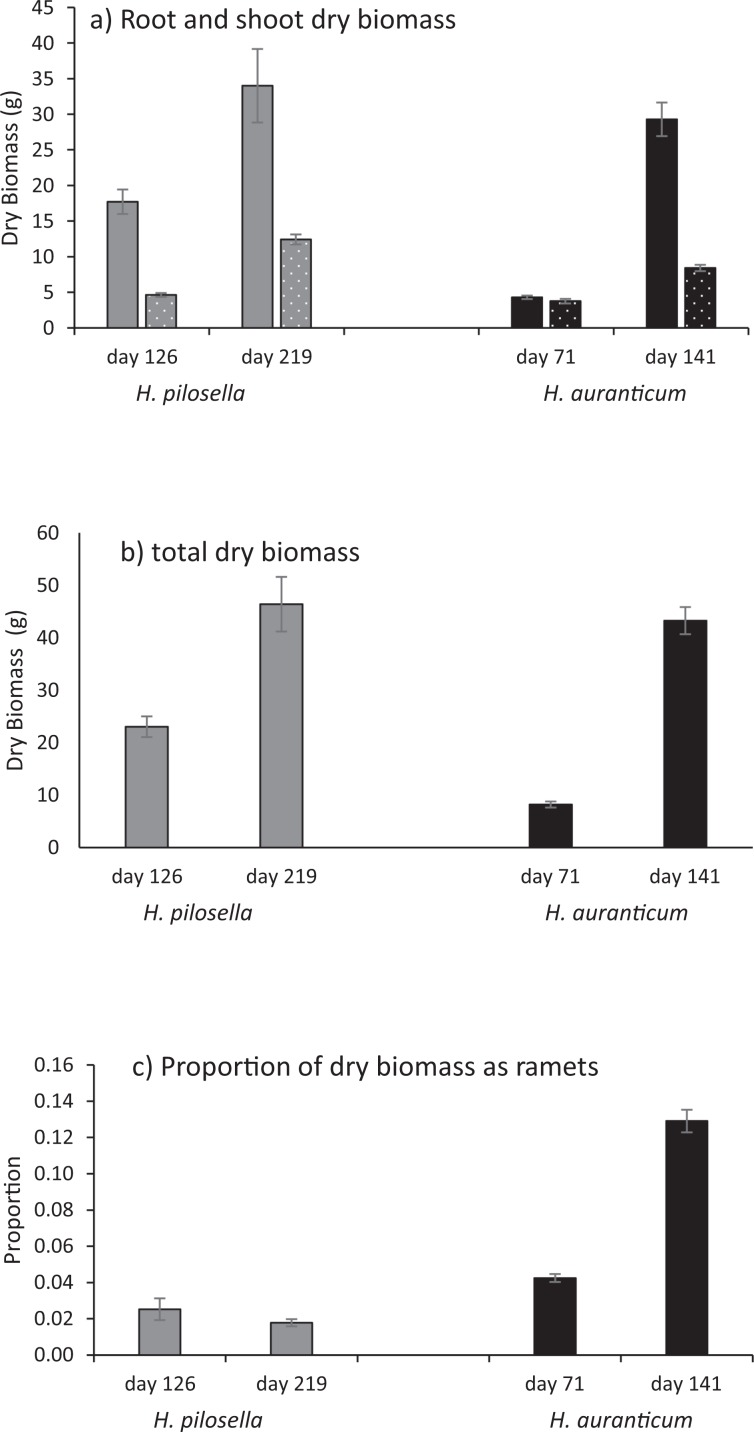
(**a**) Final average dry biomass per plant (±s.e.) of roots (solid) and shoots (pitted), (**b**) average total dry biomass including ramet weights, (**c**) proportion of total dry biomass that is new ramets, of older ramets for *H. pilosella* and *H. aurantiacum* harvested at about halfway through experiment and at final harvest (days on y axis).

For the older plantlets, both species produced about 300 leaves per plantlet, but the time taken for this varied dramatically. Ramets of *H. aurantiacum* began with an average of 12.3 ± 9.1(s.d.) leaves (range 5–29) and those of MEHW began with 10.3 ± 6.2(s.d.) leaves (range 4–28). For *H. aurantiacum*, plantlets grew approximately 300 leaves over the late summer and autumn period (2.1 leaves per day), while *H. pilosella* plantlets didn’t grow this many leaves until the following spring (overall 1.4 leaves per day; Fig. 7). Furthermore *H. aurantiacum* added an average of nearly 50 stolons per plant by the beginning of winter while *H. pilosella* had a slower and lower production of ramets; in early spring *H. pilosella* plantlets had produced nearly 20 stolons per plant (Fig. 8). *H. aurantiacum* stolons were 21.4 ± 10.9(s.d) % of the total dry biomass yielding an overall allocation to vegetative reproduction of 25.7 ± 11.7(s.d) % of the total biomass (stolons plus ramets). Plants of both species started producing new stolons after about 40 days. For both species dry root biomass was at least double that of the biomass of both leaves and stolons (Fig. 9). The proportion of weight produced as ramets was only greater than 10% for *H. aurantiacum* (Fig. 9).

## Discussion

Orange and mouse-ear hawkweed grew very quickly under the conditions provided in this experiment. Both species put considerably more into below-ground growth relative to above ground, even when ramet production was included. Below-ground capacity is very important in resource-poor habitats^22–24^ and strong below ground growth may increase resilience during snow covered winter periods, although no losses in leaves occurred at below freezing temperatures. Both species are likely to rely heavily, in the field, on the development of below ground biomass to aid competition and survival. Weigelt *et al*.^25^ found that competition occurred largely below ground in *H. pilosella* although it was less competitive under water limitation.

Invasive species are considered to be advantaged by developing strong growth in invaded ranges^16,17^ although field measurements are rare^26,27^. Invading plants would be expected to develop improved competitive attributes (EICA hypothesis^28^), however invaded range features may influence this. Rates were much quicker in our experiments than those measured in the field, perhaps associated with the well watered and non competitive conditions in this experiment. Thus, they more closely represent intrinsic growth rates. In the field in New Zealand, root:shoot ratios for *H. pilosella*^29^ and overall growth rates were much lower^25^, suggesting competition is an important modifier. Certainly *H. pilsosella* tended to colonise poor quality and degraded soils in New Zealand^30^ and in native regions^31^ which reflects the conditions in the invaded area in Australia. It is known to influence resource acquisition in native species^2^. Together with our results, this suggests that hawkweeds have the capacity for fast growth in the invaded range under conditions of higher nutrients and lower competition.

We found a large difference between the two species in the allocation of resources to vegetative reproduction which were only partially explained by the different altitudes in which the two species have invaded in Australia. *H. aurantiacum* put significantly more resources into ramets, allocating up to between 4–15% of biomass depending on age. Stolon production began when seedlings were around 4 weeks at termperatures mimicking 1000 m and at 6 weeks at 1700 m indicating a strong vegetative growth response and was a significant component of biomass. In contrast, *H. pilosella* seedlings did not produce stolons with ramets and plantlets only produced stolons when plants were over 1 year old and probably closer to two years old. The rate of production of stolons was slow (2.6 stolons per month), in contrast to 9.8 stolons per month for *H. aurantiacum*. However, rates of stolon production in *H. pilosella* was about twice that recorded further south in Victoria (~5.5 stolons per month^32^) and greater than field plantlets in New Zealand (1.6 stolons per plantelet^33,34^). In New Zealand mouse-ear and king devil HW relied almost exclusively on clonal growth for population growth but clearly new incursions were a result of seed dispersal^29^. Relative to king devil HW, *H. pilosella* produced less seed, but more daughters which established further from the parent^29^. Our results suggest that *H. aurantiacum* is even more effective in spreading from vegetative growth. Furthermore, given that neither species produced flower spikes during our experiments, it suggests that *H. aurantiacum* was a more effective invader than mouse-ear in the NSW alps, spreading through stolons and the development of weed mats.

Both species were able to maintain strong growth, except when temperatures reflected winter norms, particularly at temperatures mimicking the higher altitude, when both species did not produce new leaves until temperatures came above zero. After the first five months of growth, seedlings had produced leaves at a rate of about 0.5 leaves day^−1^. Older plantlets grew more quickly; plantlets of *H. aurantiacum* producing 2.1 leaves day^−1^ from late summer - winter temperature settings where *H. pilosella* was 3 times slower (0.7 leaves day^−1^) for the same period but it dramatically increased production when temperatures became wamer, and overall produced 1.4 leaves day ^−1^. The increase in growth rate of established plants, suggests that, in order to achieve eradication, there is a window of opportunity of about 18 months prior to flowering and before a rapid increase in growth rate when invasion patches can be eradicated before creating significant breaks in native plant cover.

*Hieracium pilosella* had seeds with better germinability than *H. aurantiacum*, although neither were as high as measured by Bear *et al*.^32^ who found up to 89% germination for 3 month old, dry stored seeds. Better germination may be important if *H. pilosella* has greater allocation to reproduction rather than vegetative spread^18^. *Hieracium pilosella* benefits more from increased sexual reproduction in its invaded range than in native range^18^. Beckman *et al*.^18^ only found 7% germination for *H. pilosella* in Germany. In NZ the high density of seed fall produced by *H. pilosella*, coupled with high seedling survival was thought to be important in the invasion of this species^29,33^. Seeds were equally viable with age, indicating a longer term seed bank may be able to develop. Further research on longevity in the field would be beneficial.

*Hieracium aurantiacum* may well be able to germinate at lower altitudes but hot conditions experienced over the summer in these more coastal areas, may well limit the probablity of survival. About 10% of *H. aurantiacum* seeds germinated at temperatures mimicking low altitudes, less than at lower temperatures mimicking higher altitudes, but seedlings did not survive the hot conditions experienced during the experiment. Despite this susceptibility, a cooler summer may allow establishment of seedlings and it is clear from this experiment that growth will be rapid. Once established, plants of both species appear to be resilient to hotter conditions. The hotter conditions experienced during the failure of the growth cabinets at day 146 (up to 24 °C) for *H. pilosella*, and the very hot conditions (>35 °C) for *H. aurantiacum* at 1000 m had no visible effect on either species.

This study has shown the enormous capacity for growth of these two species of invading hawkweeds in the alpine regions in NSW, Australia. Vegetative reproduction is critical in the early stages of invasion to occupy space. *H. aurantiacum* was far more effective in vegetative growth compared to *H. pilosella*, suggesting its rate of spread will be much greater than *H. pilosella*. Both species, once established, appear to have significant mechanisms for tolerance derived from a large below ground biomass but also an apparent ability to easily cope with heat events when adequately watered. Further work is needed to understand hawkweed responses to interactions between temperature and other variations in climate that vary with altitude (rain/snowfall, wind etc). Understanding the negative effects of competition and nutrient limitation in the field in these species will greatly enhance our capacity to model spread in these species.

## Data Availability

Data are available at 10.5061/dryad.d2547d7zr.
